# COX-2 Inhibitors, Aspirin, and Other Potential Anti-Inflammatory Treatments for Psychiatric Disorders

**DOI:** 10.3389/fpsyt.2019.00375

**Published:** 2019-05-31

**Authors:** Norbert Müller

**Affiliations:** ^1^Department of Psychiatry and Psychotherapy, Ludwig-Maximilians-Universität Munich, Munich, Germany; ^2^Marion von Tessin Memory Center, Munich, Germany

**Keywords:** Inflammation, major depression, psychoneuroimmunology, COX-2 inhibition, aspirin, psychiatry, schizophrenia

## Abstract

Inflammatory processes associated with persistent (chronic) infection have long been discussed as etiological factors in psychiatric disorders. Studies have found that people with major depression have higher levels of pro-inflammatory cytokines, for example, IL-1, IL-6, and tumor necrosis factor-alpha, and C-reactive protein. In schizophrenia, many reports have described raised levels of cytokines, for example, IL-6; and meta-analyses have confirmed these findings. Microglia cells are important in inflammatory processes, and positron emission tomography studies have shown microglia activation in both depression and schizophrenia.As a consequence of the above findings, immunomodulation is widely discussed as a potential treatment approach in both major depression and schizophrenia. The COX-2 inhibitor celecoxib was found to have a significant positive effect on major depression, not only in single studies but also in meta-analyses. Celecoxib has also been studied in schizophrenia and has shown efficacy, in particular, in early disease stages. The mixed COX inhibitor aspirin (acetylsalicylic acid) seems to have both protective and therapeutic effects on schizophrenia.This paper discusses the hypothesized role of inflammation in major depression and schizophrenia, including markers of inflammation; pertinent studies on celecoxib and aspirin; and additional immunomodulatory therapeutic strategies.

## Introduction

Studies in animal models have provided evidence that both early infection and immune activation can affect several neurodevelopmental processes, including serotonergic (1) and dopaminergic and glutamatergic neurotransmission (2, 3). Human studies on infections (4) and a cohort study on bacterial infections (5) also indicated that infection and activation of the immune system are associated with neurodevelopment. Furthermore, studies found that higher cytokine and C-reactive protein (CRP) levels in childhood increased the risk to develop depression (6) and schizophrenia (7). In meta-analyses, cytokine levels were altered in adults with major depression (MD), schizophrenia, and bipolar disorder (8, 9).

## Major Depression

### Inflammation and Depression Risk

A population-based, prospective cohort study in Denmark followed a total of 3.56 million people for 24 years and found that those who were hospitalized for an infection or visited a hospital for treatment of an autoimmune disease had a significantly higher risk for developing a depressive disorder (10). If people were hospitalized for infection, the risk increased by 62% [incidence rate ratio (IRR) 1.62]; and if they visited the hospital because of an autoimmune disease, it increased by 45% (IRR 1.45). People with a history of both risk factors had an even higher risk of developing mood disorders (IRR 2.35), indicating that the two factors interact. In this study, the risk for later mood disorders was lower after infections of the central nervous system (CNS; IRR 1.65) than after peripheral infections, for example, hepatitis (IRR 2.82) and sepsis (IRR 2.06). Interesting findings were that the shorter the time since the infection, the greater the risk of developing a mood disorder and that the highest risk was found in the 12 months after the infection (IRR 2.70). If the study had assessed all kinds of infections—not only those for which people visited a hospital—the risk of developing a mood disorder might have been even higher (10).

Another Danish population-based register study evaluated whether the use of anti-inflammatory agents, including aspirin (acetylsalicylic acid [ASA], an inhibitor of both COX-1 and COX-2), is associated with a lower rate of depression (11). The study found a dose-dependent risk; that is, continued use of low-dose aspirin reduced the risk for incident depression, whereas the use of non-steroidal anti-inflammatory drugs (NSAIDs) and high-dose aspirin increased the rate.

### Anti-Inflammatory Treatment Approaches in Major Depression

#### Non-Steroidal Anti-Inflammatory Drugs

NSAIDs act by inhibiting COX-1 and COX-2. These cyclooxygenase enzymes are necessary for the synthesis of some of the prostaglandins involved in inflammation. COX-2 inhibitors have direct effects on the CNS serotonergic system and can also affect it indirectly *via* immune processes in the CNS. A study in rats showed higher serotonin levels in both the frontal and temporoparietal cortices after administration of rofecoxib, a COX-2 selective NSAID (12). Consequently, the authors hypothesized that COX-2 inhibitors may have antidepressant effects. In a study in bulbectomized rats (a model for depression), chronic administration of the COX-2 inhibitor celecoxib decreased cytokine levels and changed the animals’ behavior (13). Brunello et al. (14) studied ASA in rats with the chronic escape deficit model and found that ASA accelerated the antidepressant effect of fluoxetine. In people with MD, a randomized, double-blind pilot study compared reboxetine plus celecoxib with reboxetine plus placebo and found a significant therapeutic effect of celecoxib (15). One interesting finding of this study was that the kynurenine/tryptophan ratio, which reflects the activity of indoleamine 2,3-dioxygenase (IDO), a pro-inflammatory, cytokine-driven enzyme, predicted the antidepressant response to celecoxib; that is, celecoxib had better effects on patients with a high level of IDO activity (16). A double-blind, randomized controlled trial (RCT) in MD (*n* = 50 patients) compared fluoxetine plus celecoxib with fluoxetine plus placebo and found a significantly better outcome in the group receiving adjunctive celecoxib (17). Similar results were found in two studies of sertraline plus celecoxib or placebo in MD (*n* = 40 and *n* = 30) (18, 19), where Hamilton Depression Rating Scale scores decreased significantly more in the celecoxib group; in one of the studies (18), serum IL-6, a pleiotropic immune-activating cytokine that primarily promotes innate and B- and T-cellular immunity and plays an important role in inflammation, correlated with the decrease in the depression rating score.

The efficacy of adjunctive treatment with an NSAID in MD was evaluated in a meta-analysis of four celecoxib studies in a total of 150 patients (20). The analysis concluded that celecoxib may be a potential treatment in this disorder, although the authors stated the benefit and safety of celecoxib and other NSAIDs need to be confirmed in larger studies of longer duration (20).

The findings of another meta-analysis on inflammation-related therapeutic approaches in MD are also of great interest (21). This analysis evaluated data from 14 studies (10 on NSAIDs, *n* = 4,258; 4 on cytokine inhibitors, *n* = 2,004) and found that the anti-inflammatory treatments had positive effects compared with placebo [standardized mean difference (SMD), −0.34; 95% confidence interval (CI), −0.57 to −0.11; *I* ^2^ = 90%], both in depression (SMD, −0.54; 95% CI, −1.08 to −0.01; *I* ^2^ = 68%) and in depressive symptoms (SMD, −0.27; 95% CI, −0.53 to −0.01; *I* ^2^ = 68%). The type of depression (clinical depression vs. depressive symptoms) or the agent (NSAID vs. cytokine inhibitor) did not explain the heterogeneity of the studies with respect to differences in treatment regimens and patient populations. Sub-analyses provided support for the positive effects of celecoxib (SMD, −0.29; 95% CI, −0.49 to −0.08; *I* ^2^ = 73%) on both remission [odds ratio (OR), 7.89; 95% CI, 2.94 to 21.17; *I* ^2^ = 0%] and response (OR, 6.59; 95% CI, 2.24 to 19.42; *I* ^2^ = 0%). Six of the studies reported adverse effects but found no difference in the rate of gastrointestinal or cardiovascular side effects at 6 weeks or infections at 12 weeks between the active treatments and placebo. The meta-analysis suggested that treatment with an anti-inflammatory agent, particularly celecoxib, can ameliorate symptoms of depression but does not carry a higher risk of side effects (21). Other studies, however, reported higher rates of adverse cardiovascular events with COX-2 inhibitors (22).

ASA has also been studied in depression. In an 8-week RCT in MD, patients were assigned to 160 mg of aspirin add-on to sertraline (*n* = 50) or placebo add-on (*n* = 50); the groups were matched for age, gender, and severity of depression (23). After 4 and 8 weeks of treatment, depression scores were significantly lower than at baseline only in the sertraline plus aspirin group. These results indicate that aspirin has positive effects on depression, although the unusually low responder rate in the sertraline (plus placebo) group may indicate that treatment resistance affected the results. A complex, placebo-controlled study of patients with bipolar depression found a significantly higher response rate in the aspirin group; however, the comparison of the mean values found no significant difference between aspirin and placebo (24).

Another recent meta-analysis included additional studies on NSAIDs and differentiated between studies on patients with a diagnosis of MD and patients with “classical” inflammatory diseases, such as arthritis or psoriasis, who also had depressive symptoms (25). The analysis found highly significant effects on both MD and depressive symptoms and a highly significant overall effect on the combined analysis of both indications. In the analysis of different substance classes and mechanisms of action of the anti-inflammatory agents, classical NSAIDs were significantly superior to placebo. Other anti-inflammatory compounds, such as cytokine inhibitors, glucocorticoids (two RCTs), and minocycline, also showed a significant advantage than did placebo. The overall effect of all anti-inflammatory substances was *p* = 0.00001.

**Table 1** gives an overview of studies on selective COX-2 inhibitors and ASA in MD. Despite the limitations of the studies described above, they provide important information on the effects of anti-inflammatory treatment and, in particular, COX-2 inhibition in MD. Additional studies are needed in larger samples. Furthermore, studies need to consider the high placebo response, particularly in add-on studies with an effective antidepressant, and the severity of depression.

**Table 1:** Clinical studies of selective COX-2 inhibitors and the mixed COX-1/COX-2 inhibitor acetyl salicylic acid (ASA) in major depression.

Authors	Diagnosis	Duration of trial	N	Study design	Concomitant drug	COX-2 inhibitor	Outcome
Abbasi et al. (18)	Major depression	6 weeks	40	Randomized, double-blind, placebo-controlled	Sertraline 200 mg	Celecoxib 400 mg/day	Significantly better response and remission rates in celecoxib group
Akhondzadeh et al. (17)	Major depression	6 weeks	40	Randomized, double-blind, placebo-controlled add-on	Fluoxetine (flexible dose)	Celecoxib 400 mg/day	Significant superiority of celecoxib
Begemann et al. (26)	Bipolar depression, rapid cycling	>5 months	1	Open	Not specified	Celecoxib 400 mg/day	Significant improvement of depressed and manic symptoms
Castillo et al. (27)	Bipolar depression	8 weeks	41	Randomized, double-blind, placebo-controlled	Escitalopram 20 mg/day	Celecoxib 400 mg/day	Celecoxib showed significantly better response and higher remission rate
Collantes-Estevez and Fernandez-Perez (28)	Depressive syndrome, comorbid to osteoarthritis	Mean 33 days	343 (with depressive syndrome)	Open	Not specified	Rofecoxib 12.5 or 25 mg/day	Significant reduction of self-reported depression
Majd et al. (19)	Major depression	8 weeks	30 (women only)	Randomized, double-blind, placebo controlled	Sertraline (25–50 mg)	Celecoxib 200 mg/day	Significant superiority of celecoxib after 4 weeks; no difference after 8 weeks
Muller et al. (15)	Major depression	6 weeks	40	Randomized double-blind, placebo-controlled, add-on	Reboxetine (flexible dose)	Celecoxib 400 mg/day	Significant superiority of the COX-2 inhibitor
Nery et al. (29)	Bipolar disorder, depressive or mixed episode	6 weeks	28	Randomized, double-blind, placebo-controlled	Mood stabilizer or atypical antipsychotics	Celecoxib 400 mg/day	Significant superiority after 1 week, no difference at end-point
Savitz et al. (24)	Bipolar depression	6 weeks	99	Randomized, double-blind, placebo-controlled	Minocycline (200 mg/day)	ASA 162 mg/day	Main effect of ASA on treatment response
Sepehrmanesh et al. (23)	Major depression	8 weeks	100	Randomized, double-blind, placebo-controlled	Sertraline (50–200 mg)	ASA 160 mg/day	Significantly greater reduction in Beck Depression Inventory scores after 4 and 8 weeks

#### Drugs Targeting Cytokines

Tumor necrosis factor-alpha (TNF-a) promotes the activation of the innate and adaptive immune response and is a key molecule in inflammation. The anti-TNF-a antibody infliximab prevents the cytokine TNF-a from interacting with receptors on the cell surface and has an anti-inflammatory effect. TNF-a was initially designed as a treatment for inflammatory joint disorders and psoriasis but was then found to significantly improve symptoms of depression in patients with psoriasis (30).

In a random-effect meta-analysis of seven placebo-controlled RCTs, anti-cytokine treatment had significantly greater effects on depressive symptoms than had placebo (anti-cytokine drug: *n* = 1,309; placebo: *n* = 1,061; SMD = 0.40, 95% CI, 0.22 to 0.59) (31). Five of the seven studies were on anti-TNF-a agents, that is, adalimumab, etanercept, and infliximab (SMD = 0.33; 95% CI, 0.06 to 0.60). Similar, small-to-medium effect sizes for anti-cytokine therapy were obtained in the analyses of the two RCTs on adjunctive anti-cytokine treatment (SMD = 0.19; 95% CI, 0.00 to 0.37) and the eight non-randomized and/or non-placebo studies (SMD = 0.51; 95% CI, 0.34 to 0.67). The three anti-TNF-a drugs and the anti-IL-6 antibody (tocilizumab) significantly improved symptoms of depression. A meta-regression showed that baseline symptom severity was a predictor of antidepressant effect (*p* = 0.018), but sex, age, study duration, and improvement in the primary physical illness were not. An important limitation of these studies was that besides having an inflammatory disease, such as atopic dermatitis, psoriasis, Crohn’s disease, or rheumatoid arthritis, the patients had concomitant symptoms of anxiety or depression or both. In addition, their overall symptoms of depression were mild to moderate, and they did not have a formal diagnosis of depression.

To my knowledge, only one 12-week, placebo-controlled study has evaluated an anti-TNF-a antibody in treatment-resistant MD (*n* = 60). The participants, who were either taking an antidepressant (*n* = 37) or partly medication free (*n* = 23), received three infusions of infliximab (*n* = 30) or placebo (*n* = 30). Infliximab was not superior to placebo, but the study found a significant interaction between time, treatment, and baseline levels of CRP (≤5 mg/L); that is, the response rate to infliximab (62%) was higher than that to placebo (33%) in patients who had higher CRP levels at baseline. In addition, participants who responded to infliximab had significantly higher baseline concentrations of TNF-a, sTNFR1, and sTNFR2 (*p* ≤ 0.01) and significantly larger decreases in CRP (*p* ≤ 0.01) than had those who did not respond (32). This study is encouraging, in particular because treatment-resistant patients with MD represent a negative selection for treatment outcome, and it indicates that CRP—the most widespread clinical marker for inflammation—may be a biomarker of anti-TNF-a antibody treatment outcome.

#### Drugs Targeting the IL-6 Complex

As mentioned above, studies have found that in patients with depression, IL-6 levels in the peripheral blood and cerebrospinal fluid (CSF) are higher. Consequently, the IL-6 complex has been proposed as a target for anti-cytokine treatment. The above-mentioned random-effect meta-analysis of seven RCTs (31) included two open studies of the anti-IL-6 antibody tocilizumab (33, 34), both of which showed improvements in patients with concomitant anxiety and depression. Despite these findings, we still need valid, reliable data on the effects of anti-IL-6 treatment in MD and the correct target for such treatment. Because higher levels of IL-6 are found in the CSF than in the blood and in patients with depression than in controls, IL-6 in the CSF was hypothesized to be the most promising therapeutic target. Further support for this hypothesis was given by the finding that IL-6 is overexpressed in the pre-clinical model of chronically stressed rats (35). However, IL-6 levels in the periphery are not completely independent of those in the CSF or brain but are closely connected to them. For this reason, researchers have proposed that targeting peripheral IL-6 may be a potential treatment approach in depression (35). This proposal is supported by the findings in an animal model of depression that significantly higher serum IL-6 levels were found in learned helplessness rats, which were susceptible to chronic inescapable electric stress, than in control and non-learned helplessness rats, which were resilient to the same stress (36). Supporting evidence was also provided by the finding that in depression, serum levels of IL-6 predict patients’ response to ketamine (37). As an alternative, more promising strategy to inhibit IL-6, Maes et al. (38) proposed that administering soluble glycoprotein 130 (sgp 130), a component of the IL-6 complex, would increase inhibition of IL-6 trans-signaling and consequently maintain IL-6 receptor (IL-6R) signaling. In addition to tocilizumab, sirukumab, another monoclonal antibody to IL-6, has also been proposed as a potential treatment for depression (39). It acts on the signaling pathway of IL-6 and can inhibit its pro-inflammatory and anti-inflammatory effects; studies have shown beneficial effects of sirukumab in the inflammatory diseases lupus erythematosus and rheumatoid arthritis, among others (39).

#### Other Immune-Related Substances

Microglia cells act as macrophages in the brain and are important in inflammatory processes in the CNS. Positron emission tomography (PET) studies showed activation of microglia in MD (40, 41). Minocycline, an antibiotic, can cross the blood–brain barrier into the CNS, where it inhibits the activation of microglia. Consequently, it was proposed as a potential treatment in MD. A recent meta-analysis identified 18 clinical studies (a case report, RCTs, and open and ongoing trials) on minocycline in depression; however, only three RCTs (in a total of 158 participants) were suitable for analysis (42). The analysis observed a large, statistically significant antidepressant effect of minocycline compared with placebo, although limitations of the study included the small number and sample size of the trials included, their heterogeneity, and potential publication bias.

Although statins are primarily used to lower cholesterol, they also have direct anti-inflammatory effects (43, 44). For example, a population-based study in users of selective serotonin reuptake inhibitors (SSRIs; *N* = 872,216), *n* = 113,108 of whom were also taking a statin, found that the risk for (a relapse to) depression was clearly lower in participants using both SSRIs and statins than in those using an SSRI alone (43).

### Methodological Issues

Although a “gold standard” study in MD would be an RCT that directly compares the anti-inflammatory agent, for example, a COX-2 inhibitor or an anti-cytokine antibody, with placebo, it would not be ethical. Therefore, in almost all RCTs, the anti-inflammatory agent is administered as an adjunct to an antidepressant. As a result of this approach, the anti-inflammatory agent must have a large effect to show an advantage over the antidepressant alone or in combination with placebo. This problem is further exacerbated by the high placebo rate of up to 40% that is commonly seen in studies of antidepressants. Nevertheless, although diverse anti-inflammatory therapeutic approaches show a statistically significant beneficial effect, much further research is needed, and possible subgroups of responders and non-responders have to be identified.

It is well known that in more severe cases of depression, antidepressants show more efficacy than does placebo. This might also be true for anti-inflammatory agents. If possible, clinical studies should therefore include more severely depressed patients.

The pathology of depression is unlikely to be identical in every patient, and, consequently, an inflammatory process is probably not always present, although this hypothesis requires further research. Patients with treatment-resistant MD had higher levels of pro-inflammatory cytokines, which was taken as an indication that these patients’ depression might have an inflammatory origin (45). If a clinical study of anti-inflammatory compounds has a large proportion of patients with an inflammatory pathology, it is more likely to find a positive treatment effect. A goal for future research is to identify a valid, reliable marker for an inflammatory process in depression so that we can apply targeted anti-inflammatory treatment in those patients who would benefit from it.

## Schizophrenia

### Inflammation and the Rrisk for Schizophrenia

**Figure 1:**
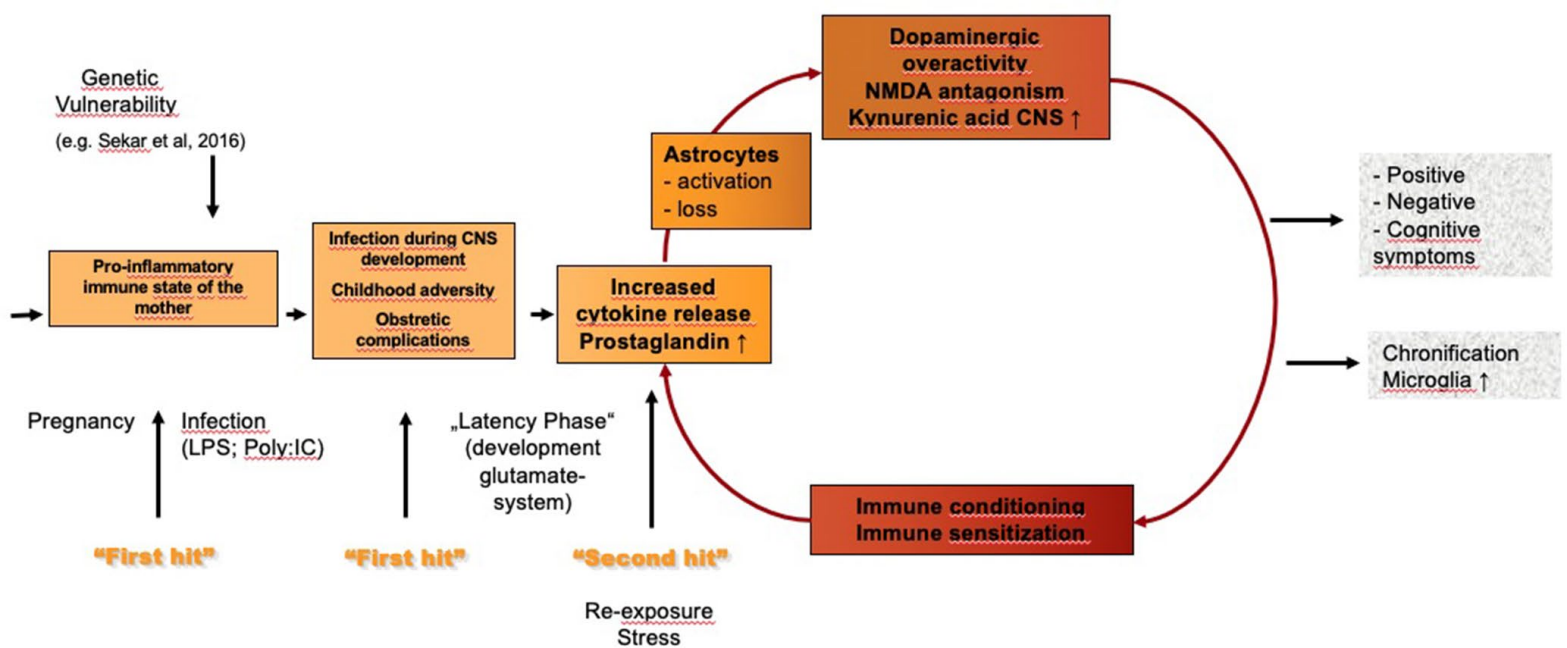
Vulnerability–stress–inflammation hypothesis of schizophrenia. LPS, lipopolysaccharides; poly:IC, polyinosinic–polycytidylic acid.

Numerous studies, including recent genetic data (46), show that an immune process and inflammation play a role in at least a subgroup of patients with schizophrenia. The results of these studies gave rise to the vulnerability–stress–inflammation hypothesis of schizophrenia (see **Figure 1**). One study in Denmark linked population-based registers nationwide and identified autoimmune disorders and severe infections that required admission to hospital as risk factors for schizophrenia and schizophrenia spectrum disorders; the risk was greatest in patients who had an autoimmune disorder and a severe infection (47). No evidence was found, however, that parental infections increased risk in the offspring (47, 48). Although the study was large, it did not have high sensitivity, and the authors consequently described the identified risk factors for schizophrenia as only the “tip of the iceberg” (48).

Another population-based study in Denmark identified all incident patients receiving antipsychotics for schizophrenia (*n* = 16,235) (49). Of these patients, *n* = 1480 (9.1%) were using concomitant NSAIDs, and *n* = 767 (4.7%) were using concomitant paracetamol. The risk of relapse was higher in the patients using NSAIDs [hazard rate ratio (HRR) = 1.21; 95% CI, 1.11 to 1.31], particularly ASA and diclofenac, but not in those using paracetamol (HRR = 0.97; 95% CI, 0.87 to 1.08). The authors analyzed subgroups of patients and found that among patients taking NSAIDs, the relapse risk was higher in those with a comorbid physical illness and lower in those who had been diagnosed with musculoskeletal disease (HRR = 0.82; 95% CI, 0.71 to 0.94) (49).

### Anti-Inflammatory Treatment in Schizophrenia

#### Non-Steroidal Anti-Inflammatory Drugs

Perhaps the most convincing evidence for an involvement of inflammation in schizophrenia is provided by the finding that anti-inflammatory medication is useful in schizophrenia (see **Table 2**). Noteworthy in this context is the paper by Sommer et al. (50), which reviews the effects of various anti-inflammatory treatments, including celecoxib, in schizophrenia.

**Table 2:** Clinical studies of the selective COX-2 inhibitor celecoxib and the mixed COX-1/COX-2 inhibitor acetyl salicylic acid (ASA) in schizophrenia.

Authors	Diagnosis	Course and duration	Duration of trial	N	Study design	Concomitant drug	Substance	Outcome
Akhondzadeh et al. (51)	Schizophrenia	Chronic type (active phase)	8 weeks	60	Double-blind, randomized placebo-controlled add-on	Risperidone (fixed dose)	Celecoxib 400 mg/day	Significant advantage of the COX-2 inhibitor
Attari et al. (52)	Schizophrenia	>2 years	6 weeks treatment follow-up after 10 weeks	60	Double-blind, randomized placebo-controlled add-on	Mixed antipsychotics	ASA 325 or 500 mg	Significant benefit of ASA after 10 weeks, better effect of higher ASA after 6 weeks
Laan et al. (53)	Schizophrenia spectrum	≤5 years	3 months	70	Double-blind randomized placebo-controlled add-on	Mixed antipsychotics	ASA 1,000 mg	Significant, larger decrease in PANSS total in ASA group
Muller et al. (54)	Schizophrenia	Not specified Mean 5.9 years	5 weeks	50	Double-blind, randomized placebo-controlled add-on	Risperidone (flexible dose)	Celecoxib 400 mg/day	Significant advantage of the COX-2 inhibitor
Muller et al. (55)	Schizophrenia	First manifestation	6 weeks	50	Double-blind, randomized placebo-controlled add-on	Amisulpride (flexible dose)	Celecoxib 400 mg/day	Significant advantage of the COX-2 inhibitor on PANS total, positive, negative, and global scores
Rapaport et al. (56)	Schizophrenia	Continuously ill Mean 20 years	8 weeks	38	Double-blind, randomized placebo-controlled add-on	Risperidone or olanzapine (constant dose)	Celecoxib 400 mg/day	No advantage on the COX-2 inhibitor
Rappard and Muller (57)	Schizophrenia	≤10 years	11 weeks	270	Double-blind, randomized placebo-controlled add-on	Risperidone (flexible dose)	Celecoxib 400 mg/day	No advantage of the COX-2 inhibitor

PANSS, Positive and Negative Syndrome Scale.

My group performed a 6-week double-blind RCT in acute schizophrenia to compare risperidone plus celecoxib with risperidone alone and risperidone plus placebo (54). Outcome was significantly better in the celecoxib add-on group (*n* = 25) than in the group receiving risperidone alone (*n* = 25) (54), particularly regarding cognition (58). Data from this study and another 6-week risperidone/celecoxib study were pooled (total *N* = 90), and the analysis found a benefit of celecoxib add-on if the illness duration was 24 months or less but no such benefit in more chronic cases. A large study that included a broad spectrum of schizophrenia patients found no advantage of celecoxib over placebo (57), whereby the disease duration of up to 10 years in many patients, some of whom had chronic schizophrenia, may have contributed to the negative result. Another study in chronic schizophrenia showed no advantage of add-on celecoxib in patients with chronic schizophrenia (56), providing further support for differential effects of COX-2 inhibitors in acute and chronic phases of the disease. To further test this hypothesis, my group performed a double-blind study of amisulpride with adjunct celecoxib or placebo in first-episode schizophrenia (55); the celecoxib group showed more improvement than did the placebo group on the positive, negative, total, and general psychopathology scores of the Positive and Negative Syndrome Scale (PANSS) (55, 59).

Similar benefits have been found for ASA in schizophrenia spectrum disorders. A double-blind RCT studied 70 inpatients and outpatients with schizophrenia spectrum disorders treated with antipsychotics and add-on aspirin 1 g/day or placebo (53). The total and positive PANSS scores decreased significantly more in patients taking aspirin add-on than in those taking placebo, and the more altered the immune function, the greater the effect on the total PANSS score. Another double-blind, placebo-controlled study evaluated 6-week adjunctive treatment with ASA in 60 inpatients with schizophrenia (52): In addition to their antipsychotic, one group (*n* = 20) received aspirin 325 mg/day; one (*n* = 20), aspirin 500 mg/day; and one (*n* = 20), placebo. At the end of the study, the PANSS positive and negative symptom scores and general psychopathology score were significantly lower in the aspirin groups than in the placebo group.

A meta-analysis of data from *n* = 264 patients from five double-blind, randomized, placebo-controlled studies on NSAIDs (celecoxib, four studies; ASA, one study) in schizophrenia found that the drugs had significant positive effects on total symptom severity and positive and negative symptom severity (60). A meta-analysis of data from *n* = 774 patients from eight studies (celecoxib, six; ASA, two) found that the drugs had significant effects only in patients with a first manifestation of schizophrenia and not in those with chronic schizophrenia and in inpatients but not outpatients (61).

#### Other Immune-Related Treatment

PET studies have shown that microglia cells are also activated in schizophrenia (62). As mentioned in the section Other Immune-Related Substances, the antibiotic minocycline can inhibit microglia activation, and studies in animal models of schizophrenia found that minocycline improves cognition (63). Double-blind, placebo-controlled, add-on studies of minocycline in schizophrenia patients found positive effects on negative and cognitive symptoms (64), and case reports described benefits for symptoms overall (65).

Other potential anti-inflammatory agents have shown some benefit in schizophrenia. For example, a meta-analysis of 26 double-blind RCTs found significant effects for *N*-acetylcysteine and estrogen (50). Positive effects were also found for interferon-gamma (IFN-γ), a cytokine responsible for the monocytic type 1 immune response (66). IFN-γ can have severe side effects, such as hallucinations, seizures, and immunosuppression, so patients must be monitored carefully.

The initial studies on monoclonal antibodies against pro-inflammatory cytokines indicate that they may also be a potential treatment approach in schizophrenia, but more studies are needed (67).

Two interesting case reports shed light on a theoretical concept that is interesting but will probably never become an alternative for schizophrenia treatment: In one case, schizophrenia seems to have transferred by bone marrow transplantation to a leukemia patient (68); and in another contrasting case, schizophrenia was remitted by bone marrow transplantation to a patient with schizophrenia (69).

### Special Issues Regarding Anti-Inflammatory Treatment in Schizophrenia

On the basis of the above, we can conclude that anti-inflammatory agents may be beneficial in schizophrenia, but their usefulness depends on the stage of the disease; that is, their efficacy is lower in chronic than in acute schizophrenia; this difference may be associated with the neuroprogression of the disease. In schizophrenia, chronification is known to negatively impact outcome. Long-term treatment with anti-inflammatory agents has not yet been studied, although it may have more beneficial effects (51). This is the case in chronic inflammatory diseases, where short-term treatment with anti-inflammatory agents has only weak effects.

Further research is required on predictors of better response to anti-inflammatory treatment in schizophrenia. In all studies to date, response to antipsychotics was worse if levels of inflammation were higher (70, 71). The question whether the outcome of anti-inflammatory treatment is better in patients with high levels of inflammation, as was shown for anti-TNF-α treatment and celecoxib in MD (18, 32), remains open. As in MD, in schizophrenia, no markers have been identified that could help predict the outcome of treatment with anti-inflammatory agents.

Studies have shown probable non-immune-mediated effects for several NSAIDs, including COX-2 inhibitors (72), although their effects on schizophrenia are most likely due to their anti-inflammatory effects. Clearer evidence that inflammation is involved in the pathophysiology of schizophrenia is provided by studies on monoclonal antibodies, which do not affect neurotransmitters in any other way. However, further research is needed on this topic.

## Conclusion

Studies and meta-analyses showing increased levels of pro-inflammatory cytokines and CRP, for example, indicate that the development of some psychiatric disorders, including MD and schizophrenia, may involve inflammation. Further support is provided by the positive effects of immunomodulatory agents in treating these disorders. Statistically significant therapeutic effects have been shown for the COX-2 inhibitor celecoxib in MD and early-stage schizophrenia, and protective and therapeutic effects have been shown for the mixed COX-1 and COX-2 inhibitor ASA (aspirin) in schizophrenia. Statistical significance, however, does not necessarily equate to clinical significance and high effect sizes. Therefore, further studies are warranted, including stand-alone studies with immunomodulators. A further limitation is that studies of these compounds in psychiatric indications lasted only several weeks, and these agents have not been evaluated in long-term studies. Celecoxib is known to be associated with an increase in cardiovascular side effects after treatment lasting about 18 months. ASA and, to a lesser extent, COX-2 inhibitors can have gastrointestinal side effects, including gastrointestinal bleeding. Nevertheless, celecoxib and, in particular, ASA are well described and, in general, are well tolerated, in a dose-dependent manner, in many different clinical indications. Another limitation is that no specific dose–response relationship studies have so far been performed in psychiatric indications, and doses have been chosen on the basis of those used in non-psychiatric indications. Different dose regimens may optimize the benefits of these substances in psychiatric indications. Many other interesting substances, such as anti-cytokines, anti-Il-6 complex substances, minocycline, and statins, that target various components of the immune system may be beneficial in MD or schizophrenia or both.

## Author Contributions

NM is the sole author of this review and performed all tasks related to preparation of the manuscript.

## Funding

The work was supported by the Foundation “Immunität und Seele.”

## Conflict of Interest Statement

The author declares that the research was conducted in the absence of any commercial or financial relationships that could be construed as a potential conflict of interest.
